# Adult Gliomas: From Molecular Insight to Clinical Horizons

**DOI:** 10.3390/curroncol32120659

**Published:** 2025-11-24

**Authors:** Giuseppe Lombardi, Valentina Baro, Andrea Landi

**Affiliations:** 1 Department of Oncology, Oncology 1, Veneto Institute of Oncology IOV-IRCCS, 35128 Padua, Italy; 2 Academic Neurosurgery, Department of Neuroscience, University of Padova, 35128 Padova, Italy; valentina.baro@aopd.veneto.it (V.B.); andrea.landi@unipd.it (A.L.)

Over the past decade, the management of adult gliomas has been reshaped by profound scientific and clinical advances. The integration of molecular biomarkers, such as IDH mutation, 1p/19q co-deletion, TERT promoter status, and MGMT methylation, has transformed these tumors from purely histological entities into biologically distinct diseases with specific prognostic and therapeutic implications [1]. This paradigm shift, crystallized in the 2021 WHO classification, has refined diagnostic precision and opened new avenues for personalized treatment. Yet, despite these achievements, adult gliomas remain among the most challenging malignancies in neuro-oncology, with an urgent need to translate biological understanding into durable clinical benefit and improved quality of life.

Surgical and radiotherapeutic innovations have evolved in parallel with molecular discoveries. Image-guided techniques, intraoperative mapping, and functional MRI have enabled more extensive resections while preserving neurological function. Likewise, radiotherapy has become increasingly conformal and biologically adaptive, improving local control and minimizing toxicity [2,3]. Meanwhile, systemic therapy continues to progress at a slower pace. The current standard of maximal safe resection followed by radiotherapy and, in many cases, chemotherapy, remains the cornerstone of management. However, emerging approaches such as IDH inhibition, targeted therapies, and novel immunotherapeutic combinations are beginning to redefine the therapeutic landscape [4].

Among the most important recent advances is vorasidenib, a dual inhibitor of mutant IDH1 and IDH2 capable of crossing the blood–brain barrier. In the phase III INDIGO trial, vorasidenib achieved a median progression-free survival of 27.7 months versus 11.1 months with placebo (HR 0.39; *p* < 0.001) in adults with residual or recurrent grade 2 IDH-mutant gliomas [4]. It also significantly delayed the time to next intervention, offering the first effective targeted systemic therapy for this molecular subgroup. Although long-term survival data are pending and the optimal timing of therapy remains under discussion, the favorable safety profile and disease-control benefit mark a turning point in the management of low-grade gliomas.

In parallel, inhibition of the MAPK pathway represents a promising but still selective opportunity. BRAF V600E-mutant gliomas, though uncommon in adults, have demonstrated meaningful clinical responses to BRAF and MEK inhibitors, both alone and in combination. Experiences with agents such as vemurafenib, dabrafenib, and encorafenib, particularly when combined with MEK inhibition, have shown antitumor activity in pleomorphic xanthoastrocytoma, ganglioglioma, and a small subset of glioblastomas. These findings, together with broader access to molecular profiling, highlight how precision oncology is progressively entering the field of primary brain tumors.

Multikinase inhibitors have also emerged as a therapeutic avenue for recurrent disease. The phase II REGOMA trial demonstrated a modest but statistically significant survival benefit of regorafenib over lomustine in recurrent glioblastoma [5], suggesting the potential role of anti-angiogenic and microenvironment-modulating therapy in selected patients. The recently presented REGOMA-2 study, discussed at the European Society for Medical Oncology (ESMO) Congress 2025, further evaluated this approach in combination with standard Stupp treatment for newly diagnosed patients with MGMT-methylated glioblastoma. Although preliminary, these results illustrate the growing interest in multitargeted strategies that may complement, rather than replace, alkylating chemotherapy.

Another emerging development involves strategies to enhance drug delivery across the blood–brain barrier (BBB). A recent phase I/II study explored the use of a skull-implantable ultrasound device (SonoCloud-9) with microbubbles to transiently disrupt the BBB in patients with recurrent glioblastoma, enabling greater local drug penetration of agents such as albumin-bound paclitaxel and carboplatin [6]. The procedure proved feasible, safe, and reproducible, suggesting that BBB modulation could become a viable adjunct to systemic therapy. Although still experimental and limited to high-grade gliomas, this approach represents a critical translational step, potentially extending the reach of agents previously constrained by poor CNS penetration.

The field is also turning toward cellular and immune-based therapies. Early-phase studies of CAR-T cells targeting EGFRvIII, IL13Rα2, and GD2 have demonstrated biological activity and occasional radiographic responses, though durable efficacy remains elusive. Next-generation CAR constructs, regional delivery, and combinatorial strategies with checkpoint inhibition are under active investigation to enhance persistence and trafficking across the BBB [7]. Collectively, these efforts underscore the transition from non-specific cytotoxic therapy toward a biologically rational, integrated framework for glioma treatment.

As outcomes improve for selected patient subsets, new challenges emerge. Cognitive preservation, neurocognitive rehabilitation, and psychosocial well-being are now integral components of care [8]. Yet, major knowledge gaps remain. Considerable heterogeneity persists even within molecularly defined classes, and predictive biomarkers of treatment response are lacking. The optimal integration of surgery, radiotherapy, and systemic therapy remains debated, as does the balance between maximizing tumor control and maintaining neurological function. Furthermore, structured survivorship pathways addressing neurocognitive sequelae, quality of life, and caregiver support are still inconsistently implemented.

The contributions included in this Special Issue, “Treatment for Glioma: Retrospect and Prospect,” offer a comprehensive and multidisciplinary overview of current advances in adult glioma management. Among the surgical papers, Morello et al. (Contribution 1) compared the transcortical and transsylvian approaches in 58 patients with insular high-grade gliomas, showing that the transcortical route—supported by intraoperative mapping—achieves higher gross total resection rates without increasing morbidity. Similarly, Ius et al. (Contribution 2) conducted a systematic review and meta-analysis on the management of adult brainstem gliomas, demonstrating that more extensive resections confer a survival advantage but at the cost of higher complication rates, underscoring the delicate balance between radicality and function. From a molecular and translational perspective, Joyce et al. (Contribution 3) reviewed the emerging role of CD133 as a driver of stemness and treatment resistance in glioblastoma, consolidating the concept that glioma recurrence is sustained by therapy-resistant subclones. In the same vein, Jezierzański et al. (Contribution 4) summarized two decades of experience with temozolomide, reaffirming its centrality in glioblastoma therapy while highlighting the persistence of resistance mechanisms linked to MGMT and DNA repair pathways. Focusing on rare molecular contexts, Garbin et al. (Contribution 5) described NF1-associated glioblastoma as a distinct biological and clinical entity, potentially characterized by a more indolent course and better outcomes than sporadic GBM, emphasizing the need for genotype-tailored management. Complementing these advances, Fischl et al. (Contribution 6) provided a prospective evaluation of quality of life and treatment satisfaction among patients with IDH-wild-type gliomas and their caregivers, revealing the predominant influence of functional status on patient-reported outcomes and highlighting the importance of supportive and psycho-oncological care. Together, these studies exemplify the multifaceted progress occurring in neuro-oncology, from surgical precision and molecular understanding to patient-centered outcomes, while also reinforcing the notion that the future of glioma management will depend on integrating biological insight with individualized, function-preserving therapy.

Looking ahead, future research should focus on developing integrated multimodal treatment frameworks that combine molecular precision with functional preservation. Large-scale, real-world registries and multicenter collaborations are needed to capture treatment patterns and patient-reported outcomes. Clinical trials should incorporate patient-centered endpoints, neurocognitive function, and quality of life alongside traditional survival measures. Equitable access to new agents and clinical research opportunities must also remain a shared goal across healthcare systems.

Adult gliomas continue to represent a formidable challenge but also a field of remarkable scientific vitality. This Special Issue offers not only a synthesis of current progress but also a roadmap for the future. Our hope is that this research will inspire continued collaboration among clinicians, scientists, and patients to bridge molecular discovery with compassionate, patient-focused care.
